# A Decision Support System to Guide Grower Selection of Optimal Seeding Rates of Wheat Cultivars in Diverse Environments

**DOI:** 10.3389/fpls.2020.00779

**Published:** 2020-06-10

**Authors:** Jordan D. Stanley, Grant H. Mehring, Jochum J. Wiersma, Joel K. Ransom

**Affiliations:** ^1^Department of Plant Sciences, North Dakota State University, Fargo, ND, United States; ^2^WestBred, Bayer CropScience, Fargo, ND, United States; ^3^Department of Agronomy and Plant Genetics, University of Minnesota, Crookston, MN, United States

**Keywords:** seeding rate, decision support system, modeling, straw strength, tillering capacity, maximum yield, decision tree

## Abstract

Seeding rate in hard red spring wheat (HRSW; *Triticum aestivum* L.) production impacts input cost and grain yield. Predicting the optimal seeding rate (OSR) for HRSW cultivars can eliminate the need for costly seeding rate research and growers using OSRs can maximize yield and seeding efficiency. Data were compiled from seeding rate studies conducted in 32 environments in the Northern Plains United States to determine the OSR of HRSW cultivars grown in diverse environments. Twelve cultivars with diverse genetic and phenotypic characteristics were evaluated at five seeding rates in 2013–2015, and nine cultivars were evaluated in 2017–2018. OSR varied among cultivar within environments. Cultivar *x* environment interactions were explored with the objective of developing a decision support system (DSS) to aid growers in determining the OSR for the cultivar they select, and for the environment in which it is sown. A 10-fold repeated cross-validation of the seeding rate data was used to fit 10 decision tree models and the most robust model was selected based on minimizing the value for model variance. The final decision tree model for predicting OSR of HRSW cultivars in diverse environments was considered the most reliable as bias was minimized by pruning methods, and model variance was acceptable for OSR predictions (RMSE = 1.24). Findings from this model were used to develop the grower DSS for determining OSR dependent on cultivar straw strength (as a measure of lodging resistance), tillering capacity, and yield of the environment. Recommendations for OSR ranged from 3.1 to 4.5 million seeds ha^–1^. Growers can benefit from using this DSS by sowing at OSR relative to their average yields; especially when seeding new HRSW cultivars.

## Introduction

Genetic improvement through continued breeding efforts leads to the development of new hard red spring wheat (HRSW) cultivars that typically provide a yield advantage over cultivars released in prior years (Austin et al., 1980). Adaptations in plant growth habit, phenotypic traits, or physiological processes related to stress, are a few examples of ways that newer cultivars may provide increased yield potential over older cultivars (Austin et al., 1989; Christopher et al., 2008; Reynolds et al., 2012). Growers have shown preference for newer cultivars, primarily driven by the opportunity for increased grain yield potential and protein content (Dahl et al., 2004). This prompts public and private seed organizations to continuously release new HRSW cultivars, resulting in the subsequent “retirement” of older cultivars. When these new cultivars are first released, they are not accompanied by a seeding rate recommendation. Growers rely on accurate recommendations for optimal seeding rates (OSR) to avoid economic losses due to uncaptured yield or excess seed waste. With the continual release of new cultivars (and subsequent discontinuation of older cultivars), growers may benefit from knowing OSR that are specific to cultivar and environment type, as this will aid growers in maximizing seeding efficiency and improve wheat yield potential.

University extension specialists commonly provide seeding rate recommendations for new cultivars based on prior seeding rate studies of cultivars released in the preceding years. After these new cultivars are subsequently tested in multi-year seeding rate studies, the actual OSR can greatly differ from the original extension recommendation. These differences can reveal 2 + years of reduced yields and economic losses due to genotype x management (GxM) interactions (Mehring et al., 2020). Although this reinforces the importance of proper seeding rate selection, with the continued release of new cultivars (and discontinuation of older cultivars), determining OSR for each cultivar is expensive, time-consuming, and repetitive research. Furthermore, the potential for genotype x environment x management (GxExM) interactions is apparent as environment-specific factors (e.g., yield potential, annual precipitation, and seasonal temperature) impact cultivar yield, and can have an interactive effect on seeding rate (Fischer, 1985; Geleta et al., 2002; Lloveras et al., 2004). Briggs and Ayten-Fisu (1979) noted the importance of including diverse environments in seeding rate studies of new cultivars; especially as some environment and cultivar combinations favor lower seeding rates. Identifying factors that may aid in predicting OSR for new varieties can eliminate the need for costly experimentation, and help growers maximize productivity and economic return. This demonstrates the importance of exploring GxExM interactions by evaluating cultivar yield and agronomic response at different seeding rates, and in diverse growing conditions, to ensure robustness in the OSR recommendation for a cultivar.

Decision support systems (DSS) have been developed to address agricultural production problems related to soil, nutrient, and precipitation, with the objective to reduce economic losses for growers and promote sustainability by minimizing environmental impact (Bonfil et al., 2004; Wang et al., 2010). These type of systems can provide environment-specific management recommendations based on location and field-specific information provided as inputs in a computer-based algorithmic model. For example, Small et al. (2015) developed a DSS to aid growers in managing late blight disease in potatoes (*Solanum tuberosum* L.). Weather data, crop information, and grower management practices were all variables incorporated into this system that would alert growers when conditions were favorable for late blight, so growers could ensure timely management for disease prevention. Most DSS developed to date have focused on nutrient or disease management. Other DSS have been developed that are specific to crop management, but they are commonly modeled in high productivity regions (i.e., southern United States), and thereby likely to be highly-sensitive to even slight changes in input variables.

Developing a predictive model for determining OSR for new cultivars could eliminate the time lag, expense, and repetition of the current method with field trials. This type of model could be coupled with environment-specific data and incorporated into a DSS to allow for the varying effects of environmental interactions to be accounted for when determining an OSR for a new cultivar.

Regression functions (linear and non-linear) are commonly used to model agronomic responses in seeding rate studies (Geleta et al., 2002; Lemerle et al., 2004). Regression equations from these models are useful when considering yield tradeoffs relative to seeding rate changes and can also be used to determine an estimate for OSR (Wiersma, 2002). However, when these models are fit to only one set of data, predictions produced by the model can be greatly biased and parameters have large standard error (Jones and Carberry, 1994). Various methods of splitting of datasets can be used to minimize these errors when conducting statistical analyses (Crowley, 1992). A prior HRSW seeding rate study conducted in ND and MN produced regression models predictive for grain yield by dividing the original dataset into two subsets (Mehring et al., 2020). This method represents the validation set approach.

When using the validation set approach, only a portion of the dataset (training set) is used to fit a predictive model. The other portion of the dataset (validation set) is then used to test the fit of the training model. Results for this test include the root mean squared error (RMSE) value, which provides an estimate for model accuracy as it represents the test error associated with differences in predicted and observed values. Akin to using several regression functions to identify a regression model best-fit for data, comparisons among models produced by various statistical learning methods can be readily accomplished by evaluating RMSE values (James et al., 2014). This process of evaluating the accuracy (fit) of these predictive models is called model assessment. Model assessment is critical for identifying and selecting the machine learning method that will best represent the data, while minimizing bias and error.

The validation approach is an efficient way to develop and test a predictive model. However, decreasing the number of observations used to train the model will inherently decrease the power of the test, increasing the likelihood of committing a Type-II error (fail to reject the null hypothesis, when the null hypothesis is false). As it is unlikely that training set data will be exactly representative of the validation set data, validation-trained models are likely to have higher RMSE values compared to models fit to only one dataset. To address these issues, cross-validation approaches are used in place of the traditional validation approach. Cross-validation is a resampling method that is used to perform multiple “model-training” iterations prior to producing a final model that is based on the average fit of these iterations. Wu et al. (2012) demonstrated the benefits of cross-validation in regression-based modeling as they noted reduced bias in predicted values and a lower RMSE value compared to one-time regression analysis. An improvement on this method can be made by dividing the original dataset, and performing multiple cross-validation iterations on each subset, then averaging these results to determine a final model. This k-fold cross-validation method is a considerable improvement on the validation approach, as it can provide for a stable, reliable predictive model. The application of the k-fold cross-validation method has been demonstrated previously in various ecological and agricultural studies (Wiens et al., 2008; Yost et al., 2018).

Numerous algorithms have been developed to guide classification of data to produce decision trees that are user friendly as they do not require extensive knowledge to interpret. In experiments with multiple levels for each independent variable, the classification and regression trees (CART) algorithm can be used to readily produce decision trees. The use of this approach was demonstrated by Waheed et al. (2006), as they applied the CART decision tree algorithm to classify experimental plots based on irrigation use, weed management, and fertilization.

The objective of this research was to develop a DSS to improve grower selection of OSR for newer HRSW cultivars sown in the varying growing environments throughout North Dakota and Minnesota. This DSS will benefit HRSW growers by providing them with a tool to promote optimal seeding efficiency and maximum yield for sustainable production.

## Materials and Methods

### Site and Experiment Description

Data from seeding rate trials conducted in North Dakota (ND) and Minnesota (MN) in the northern United States from 2013–2015 and 2017–2018 (32 total environments) were compiled for this research. Four locations were from 2013–2015 experiments at Prosper, ND and Crookston, Hallock, and Perley, MN. Two locations were from 2014 and 2015 experiments at Kimball, and Lamberton, MN. Experiment locations in 2017 and 2018 included Dickinson (2018 only), Hettinger, Minot, and Prosper, in ND, and Crookston, and Lamberton, in MN. Location and site descriptions for combined dataset are detailed in Table 1.

**TABLE 1:** Location and soil characteristics^†^ of environments in seeding rate study.

**Location^**‡**^**	**Soil series**	**Taxonomy**	**Slope (%)**
**North Dakota**			
Dickinson	Arnegard	Fine-loamy, mixed, superactive, frigid Pachic Haplustolls	0–2
Hettinger	Shambo	Fine-loamy, mixed, superactive, frigid Typic Haplustolls	0–2
Minot	Forman	Fine-loamy, mixed, superactive, frigid Calcic Argiudolls	3–6
	Aastad	Fine-loamy, mixed, superactive, frigid Pachic Argiudolls	3–6
Prosper	Kindred	Fine-silty, mixed, superactive, frigid Typic Endoaquolls	0–2
	Bearden	Fine-silty, mixed, superactive, frigid Aeric Calciaquolls	0–2
**Minnesota**			
Hallock	Northcote	Very-fine, smectitic, frigid Typic Epiaquerts	0–1
Perley	Fargo	Fine, smectitic, frigid Typic Epiaquerts	0–1
Crookston	Wheatville	Coarse-silty over clayey, mixed over smectitic, superactive, frigid Aeric Calciaquolls	0–2
Lamberton	Webster	Fine-loamy, mixed, superactive, mesic Typic Endoaquolls	0–2
	Normania	Fine-loamy, mixed, superactive, mesic Aquic Hapludolls	0–2
Kimball (2014)	Fairhaven	Fine-loamy over sandy or sandy-skeletal, mixed, superactive, mesic Typic Hapludolls	0–2
Kimball (2015)	Dakota	Fine-loamy over sandy or sandy-skeletal, mixed, superactive, mesic Typic Argiudolls	2–6
	Ridgeport	Coarse-loamy, mixed, superactive, mesic Typic Hapludolls	2–6

*^†^Soil data obtained from NRCS-USDA, 2018. ^‡^Ordered by longitude, west to east.*

The OSR was determined for each cultivar x environment combination based on regression equation output from SAS 9.4 (PROC REG). The model considered best fit for data (linear or quadratic) was determined by maximizing *R*^2^ and minimizing RMSE values. For linear fits, OSR was the seeding rate treatment at which maximum yield was observed. For quadratic fits, OSR was determined by evaluating the coefficients of the equation. Quadratic equations with a negative linear coefficient (second term) were assigned the lowest seeding rate treatment as the OSR. For all other quadratic models, the OSR was calculated by solving the first derivative of the quadratic equation.

### Data Structure

Environments and cultivars were characterized prior to modeling. Environments were characterized based on latitude and longitude (decimal degrees), planting date (d of the year), and average HRSW yield (Mg ha^–1^) observed in environment for the respective year (Table 2). These factors were selected as they can be readily determined by growers (or estimated based on field records from prior years) to be used as inputs in a DSS. The use of continuous variables to represent environments was used to minimize bias when grouping similar data across environments, and reduce model overfitting, that could increase error in OSR prediction. This also ensured models were robust, and thereby relevant to a greater number of growers.

**TABLE 2:** Location and year details for 32 environments in North Dakota and Minnesota.

**Location^**†**^ Year**	**Latitude**	**Longitude**	**Previous crop**	**Planting date**	**Harvest date**	**Yield (Mg ha^–1^)**
Dickinson, ND	46.981	−102.824				
2018			HRSW^‡^	2-May	13-Aug	3.82
Hettinger, ND	46.012	−102.647				
2017			Soybean	26-Apr	3-Aug	1.94
2018			Soybean	27-Apr	16-Aug	3.09
Minot, ND	48.180	−101.304				
2017			Soybean	21-Apr	19-Aug	1.81
2018			Soybean	3-May	8-Aug	4.31
Prosper, ND	47.003	−97.116				
2013			Soybean	16-May	22-Aug	4.69
2014			Soybean	27-May	3-Sep	4.43
2015			Soybean	9-Apr	21-Aug	4.67
2015			Soybean	22-May	25-Aug	3.62
2017			HRSW	22-Apr	21-Aug	4.51
2018			HRSW	30-Apr	31-Jul	4.22
Hallock, MN	48.802	−96.982				
2013			Soybean	16-May	3-Sep	7.27
2014			Soybean	23-May	6-Sep	5.45
2015			Soybean	16-Apr	13-Aug	5.62
Perley, MN	47.151	−96.752				
2013			Soybean	8-May	16-Aug	5.80
2014			Soybean	22-May	2-Sep	6.00
2015			Soybean	13-Apr	11-Aug	7.03
Crookston, MN	47.815	−96.616				
2013			Soybean	10-May	8-Aug	6.14
2013			Soybean	29-May	26-Aug	6.38
2014			Soybean	17-May	27-Aug	4.95
2014			Soybean	4-Jun	27-Aug	4.55
2015			Soybean	23-Apr	21-Aug	6.35
2015			Soybean	22-May	25-Aug	5.38
2017			Soybean	3-May	29-Aug	5.09
2018			Soybean	7-May	8-Aug	3.23
Lamberton, MN	44.241	−95.312				
2014			Soybean	21-Apr	20-Aug	5.14
2015			Soybean	4-Apr	12-Aug	5.62
2015			Soybean	27-Apr	12-Aug	4.55
2017			Soybean	17-Apr	23-Aug	3.69
2018			Soybean	7-May	10-Aug	2.52
Kimball, MN	45.417	−94.324				
2014			Soybean	26-Apr	14-Aug	5.54
2015			Soybean	8-Apr	31-Jul	5.97

*^†^Ordered by longitude, west to east. ^‡^HRSW, hard red spring wheat, *Triticum aestivum*, L.; Soybean, *Glycine max* (L.) Merr.*

Specific phenotypic and genetic traits were used to characterize the HRSW cultivars evaluated in this study (Table 3). Twelve cultivars were evaluated in 2013–2015 (Albany, Briggs, Faller, Kelby, Knudson, Kuntz, Marshall, Oklee, Rollag, Sabin, Samson, and Vantage) and nine cultivars in 2017–2018 (LCS Anchor, Lang-MN, Linkert, Prevail, Shelly, Surpass, SY Valda, ND VitPro, and TCG Wildfire). Data specific to each cultivar included gene expression for *Ppd-D* (photoperiod response), *Rht-B* and *Rht-D* (semi-dwarfing genes), and phenotypic characteristics for plant height, tillering capacity, straw strength (as a measure of lodging resistance), and heading date. Genotyping of the cultivars was done by the Wheat Genotyping Center at the USDA-ARS Cereal Crops Research utilizing polymerase chain reaction (PCR) methods. Agronomic measures compiled from published HRSW variety trial data from ND (NDSU, 2014–2018) and MN (Univ. of MN, 2008–2018) were used to characterize cultivars for phenotypic traits. A *Z*-score analysis approach [similar to that demonstrated by Laundre and Reynolds (1993); Ellsworth et al. (1998), and Rahman et al. (2009)] was utilized to determine cultivar tillering capacity (Stanley, 2019). Tillering capacity was based on *Z*-score standardized values from tillering evaluations of HRSW cultivars at spaced plantings by Stanley (2019); where cultivar tillering capacity rating is: High (*Z* > 0.67), Moderate (0.67 ≤ *Z* ≥ −0.67), or Low (*Z* < −0.67).

**TABLE 3:** Genetic and phenotypic characteristics of HRSW cultivars.

**Cultivar**	**Photoperiod response (*Ppd-D1*)**	**Semi-dwarf gene**	**Tillering capacity**	**Plant height^**†**^**	**Straw strength**	**Heading**
			***z*-score**	**cm**	**1 to 9^**‡**^**	**DAP^**§**^**
Albany	Insensitive	*Rht-B1*	1.33^¶^	77.0	5	63
LCS Anchor	Sensitive	*Rht-D1*	−0.23	71.9	4	58
Briggs	Insensitive	*Wild-type*	−1.03	83.3	7	57
Faller	Insensitive	*Rht-B1*	1.70	83.3	5	61
Kelby	Sensitive	*Rht-D1*	−1.10	72.6	4	58
Knudson	Sensitive	*Rht-B1*	0.63	78.0	5	60
Kuntz	Sensitive	*Rht-D1*	−0.37	75.4	4	60
Lang-MN	Sensitive	*Wild-type*	0.37	82.6	5	61
Linkert	Insensitive	*Rht-D1*	−0.83	72.9	2	59
Marshall	Insensitive	*Rht-D1*	0.73	78.2	4	63
Oklee	Sensitive	*Wild-type*	−0.80	80.5	6	58
Prevail	Sensitive	*Wild-type*	0.67	78.2	4	58
Rollag	Insensitive	*Rht-D1*	−0.73	75.9	3	59
Sabin	Sensitive	*Wild-type*	1.47	78.0	6	61
Samson	Sensitive	*Rht-B1*	−1.77	73.9	3	60
Shelly	Insensitive	*Rht-B1*	1.07	77.0	5	62
Surpass	Insensitive	*Wild-type*	−0.27	79.8	6	57
SY Valda	Insensitive	*Rht-D1*	−0.90	75.9	4	60
Vantage	Insensitive	*Wild-type*	−0.07	77.5	2	64
ND VitPro	Insensitive	*Rht-B1*	1.33	80.0	4	59
TCG Wildfire	Sensitive	*Rht-B1*	−1.20	86.6	4	60

*^†^Agronomic measures for phenotypic traits averaged from HRSW variety trial results (NDSU, 2014–2018; Univ. of MN, 2008–2018). ^‡^1–9; 1 is erect, 9 is lying flat. ^§^DAP, days after planting. ^¶^Rating based on *Z*-score analysis approach described by Stanley (2019); High, *Z* ≥ 0.67; Moderate, 0.67 ≤ Z ≥ −0.67; Low, <0.67.*

### Statistical Analysis and Model Development

Analysis and modeling were completed in R 3.5.3 statistical software (R Development Core Team, 2019) using the *caret* package (Kuhn et al., 2016). Variable independence was verified by Pearson’s correlation test prior to modeling. Highly correlated variables (*r* ≥ |0.8|) were excluded to minimize multicollinearity and overfitting of models. Various machine learning approaches were considered for use in fitting a robust model that would support a grower DSS, including ridge regression, elastic net, least absolute shrinkage and selection operator (LASSO) regression, stepwise regression, decision tree, and random forest. These techniques were considered as they have been demonstrated in numerous agronomic and production-focused studies (Williams et al., 1979; Piaskowski et al., 2016; Sharif et al., 2016; Qin et al., 2018; Ransom et al., 2019). The decision tree machine learning technique was considered the most appropriate for this study as the primary objective of this study was to develop a DSS for growers, and results from this technique were readily transferrable to a DSS. Additionally, based on prior knowledge of environment interactions with both seeding rate and HRSW cultivars (Stanley, 2019) and the diversity of wheat production environments throughout the Northern Plains region, a tree-based approach would minimize bias when determining groupings of environments in the dataset. Therefore, the methods and results of this study are focused on the decision tree algorithm utilized in *R*.

To ensure robustness in the final decision tree model, preliminary models were fit to data split into *k* random subsets, with *k*-1 subsets used as a training set, and the remaining subset withheld from the training step and used as the validation set; repeated for *k* iterations. Utilizing an approach similar to James et al. (2014), a *k*-fold repeated cross-validation was performed with two different settings for *k* (*k* = 5 and *k* = 10) to produce resampling measures for assessing models and determining tuning parameters for each model. The model with the lowest RMSE value was selected as the optimal model (Breiman et al., 1984).

Utilizing an approach demonstrated in other studies (Mohammadi et al., 2010; Hitziger and Ließ, 2014), Mallows’ complexity parameter (Cp) statistic was used in *R* to guide variable selection at each split in the decision tree to prevent overfitting of a model (Sreenivasulu and Rayalu, 2018). The variable producing the lowest Cp value at a split was selected as the primary variable at that branching point. Variable importance measures were selected for inclusion in *R* output, with variables ranked according to level of impact on OSR prediction based on the absolute value of the *t*-statistic for each model parameter (Strobl et al., 2007; Ruβ and Brenning, 2010).

## Results and Discussion

Cultivar and environment variables were considered independent, as values for Pearson’s correlation coefficient were all acceptable (*r* ≤ |0.8|). Initial models were prone to overfitting to specific latitude and longitude, so these variables were excluded from analyses. This coincides with the objective of this study, to develop a predictive model that is relevant to a broad audience of growers. Additionally, models overfit to individual locations or environments are not robust, and likely to be poor predictors of OSR for the same location in future years.

The 10-fold repeated cross-validation provided a training dataset that was most representative of the whole dataset, as the decision tree models fit by the 10-fold repeated cross-validation was more accurate at predicting OSR than models fit by the 5-fold (average RMSE of 1.250 and 1.264, respectively). This is because the additional subsets in the 10-fold provided for a more robust model, as the ratio of data comprising the training and validation sets were 316:35 samples for the 10-fold, and 281:70 samples for the 5-fold. With greater representation of cultivar and environment data in each 10-fold train set, and fewer samples in each validation set, the final decision tree model was fit after “viewing” the dataset from multiple angles.

**FIGURE 1:**
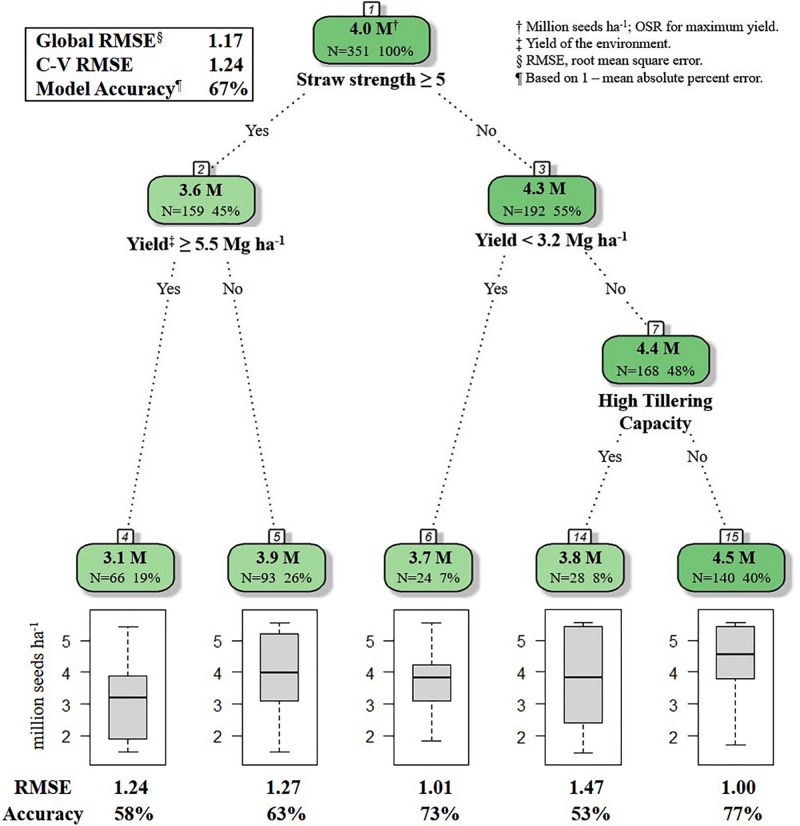
*R* decision tree model for selecting optimal seeding rate for HRSW cultivars in differing environments in ND and MN (*n* = 351). Straw strength rating (1–9; 1 is erect, 9 is lying flat) for varieties in HRSW variety trial publications from North Dakota State University Extension, 2018 and University of Minnesota, 2018. Tillering capacity determined from *Z*-score standardized values from tillering evaluations of HRSW cultivars at spaced plantings by Stanley (2019); where cultivar tillering capacity rating is: High (*Z* > 0.67), Moderate (0.67 ≤ *Z* ≥ −0.67), or Low (*Z* < −0.67). Number of samples and percent of whole dataset are reported for root, nodes and leaves. Model Accuracy = 1–mean absolute percent error.

For the decision tree algorithm, the 10-fold repeated cross-validation provided a selection of 10 decision tree models. The model selected for the final decision tree had a RMSE of 1.2386 (Table 4). As RMSE values are reported in the same units as OSR (million seeds ha^–1^), and OSR observations were recorded to three decimals in the seeding rate dataset, one may postulate that any of the models from iterations 6, 8, or 9 could have been selected for the final decision tree. To avoid bias in this decision, the final model for the decision tree was automatically selected in *R*, by including a data step for making the selection based on the iteration with the lowest RMSE value. Mallows’ Cp value used to guide variable selection (to prevent overfitting of the decision tree model) at each potential branching point was 0.0151 (Table 4). Branching ceased when all variables at a potential branch point produced a Cp value > 0.0151. The OSR at each terminal node (leaf) is the mean OSR of the data comprising that node (Figure 1).

**TABLE 4:** Modeling summary from the 10 iterations of the decision tree algorithm in *R* analyzing the seeding rate dataset (*n* = 351).

**Iteration**	**RMSE^**†**^**	**Cp**
1	1.2650	0.0057
2	1.2629	0.0060
3	1.2633	0.0063
4	1.2537	0.0077
5	1.2487	0.0083
6	1.2395	0.0097
7	1.2386	0.0151
8	1.2390	0.0187
9	1.2411	0.0433
10	1.2669	0.0734

*^†^RMSE, root mean squared error; Cp, Mallows’ complexity parameter.*

The global model from the decision tree algorithm was predictive of OSR with 67% accuracy (based on 1–mean absolute percent error). The R model output for the decision tree algorithm revealed variables impacting OSR (Figure 1). Nodes (branching points) included both phenotypic characteristics (straw strength, tillering capacity) and environment (yield of the environment). Based on variable importance measures (Pratt, 1987) reported in *R* (scaled relative to 1), the primary variable influencing OSR in the decision tree model was straw strength, with a relative variable importance of 25.7% (Figure 1). Other variables affecting OSR included yield of the environment (21.0%), tillering capacity (17.6%), and plant height (17.3%). *Rht-D* and *Rht-B* partially influenced OSR determined by the decision tree at 13.4% and 5.0%, respectively. According to the decision tree model, cultivar differences in expression for *Ppd-D* (gene for photoperiod response) did not influence OSR.

**FIGURE 2:**
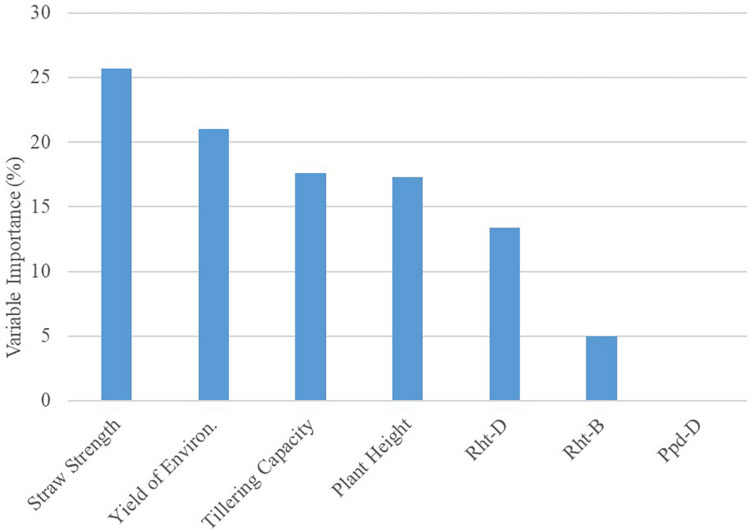
Results for Variable Importance output from decision tree model analyzing seeding rate dataset (*n* = 351) in *R*. Importance is relative to 100%.

The root node in the decision tree represented GxM influences on yield, as OSR were differentiated based on cultivar straw strength rating (Figure 1). This follows previous reportings of differences in OSR for cultivars varying in straw characteristics that affected lodging potential (Faris and De Pauw, 1980). The model also indicated GxExM interactions, as differential effects on OSR were dependent on straw strength and average yield of the environment (Figure 1). This is similar to what Otteson et al. (2007) documented for GxE interactions, where different seeding rates were considered optimal for yield. For HRSW cultivars with a favorable straw strength rating ≤4 (where 1 is best, 9 is poor), tillering capacity was a determinant of OSR, but only in environments with average yield ≥3.2 Mg ha^–1^ (Figure 1). This revealed differences in management practices that are optimal for yield due to GxE interactions (demonstrated by cultivar phenotype expression as determined by growing conditions). This is explained by the understanding that in resource-limited environments (e.g., water or nutrient deficiencies), expression of plant phenotype(s) associated with yield can be severely restricted (Richards et al., 2010; Wasson et al., 2012). This is further demonstrated by findings of Hucl and Baker (1990) for HRSW cultivars grown in semi-arid environments in Canada (average yield of 3.55 Mg ha^–1^). Though cultivars differed in tillering capacity, OSR for maximum yield was similar among cultivars in environments with average yield ≥3.2 Mg ha^–1^. Variables absent from the final decision tree were plant height and all of the genetic traits (*Rht-B*, *Rht-D*, and *Ppd-D*). However, as previously indicated, all of these variables (except *Ppd-D*) were of importance to the decision tree model, thereby of influence on OSR (Figure 2).

**FIGURE 3:**
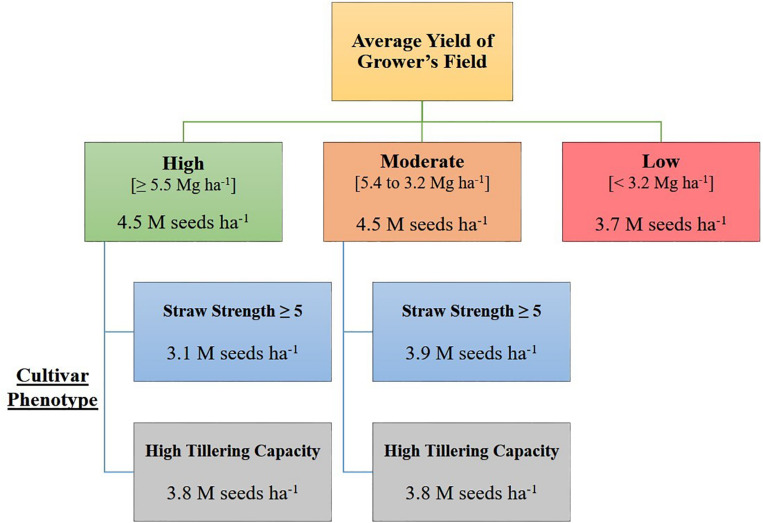
Decision support system (DSS) for growers to determine optimal seeding rates for HRSW cultivars sown in diverse yielding environments in ND and MN. Based on decision tree model in *R* from analysis of seeding rate dataset (*n* = 351).

Based on the decision tree model, growers seeding in high yielding (average yield ≥ 5.5 Mg ha^–1^), or moderate yielding (average yield 5.4 to 3.2 Mg ha^–1^) environments, should seed at a rate of 4.5 million seeds ha^–1^, unless growers are seeding a cultivar with known phenotypic characteristics requiring a lower seeding rate [i.e., poor straw strength (rating ≥ 5) or high tillering capacity] (Figure 1). Growers in low yielding environments (average yield < 3.2 Mg ha^–1^) can maximize yield by seeding HRSW at a rate of 3.7 million seeds ha^–1^ (Figure 1). In general, OSR for these environment types differentiated by average yield are similar to recommendations made by Holliday (1960) and Donald (1963), where environments with greater resource availability are expected to have higher OSR. Figure 3 was produced to provide growers with a DSS to readily determine OSR based on their selection for HRSW cultivar and the environment in which it is sown.

Though the level of variance was slightly higher for the decision tree model compared to linear regression models, the trade-off was for reduced bias in OSR predictions produced by the decision tree model. Similar to the other algorithms included in this study, the accuracy of the OSR produced by the decision tree model are greatly dependent on the data used to develop the model. This is why it was important to utilize the same resources when characterizing cultivars. Additionally, with the expectation for year-to-year variability in environmental factors (i.e., temperature, rainfall accumulation, and growing season length) influencing wheat growth in each environment, average grain yield was used to characterize environments (Slafer et al., 2014; Alvarez Prado et al., 2017). This is primarily because yield as a model parameter allows growers to readily determine OSR based on yields on their individual operations.

The recommendations outlined in the DSS improve the accuracy of predictions for OSR (Model RMSE = 1.17 million seeds ha^–1^; Cross-validation RMSE = 1.24 million seeds ha^–1^) in comparison to the current generalized recommendation of Wiersma and Ransom (2017) for 3.8 to 4.1 million seeds ha^–1^ (RMSE = 1.27 million seeds ha^–1^). However, as RMSE values for the terminal nodes (leaves) in the decision tree model ranged from 1.0 to 1.5 million seeds ha^–1^, there are apparent limitations in these findings due to the amount of error in predicted versus observed OSR values. Variability in the OSR recommendations at each terminal node could be reduced by allowing additional branching points, however, this would lead to overfitting of the decision tree model and reduce the scope of these findings. This indicates that growers should not simply default to the OSR indicated by the DSS, but rather utilize information from this tool to guide seeding rates of newer HRSW cultivars. Growers can adapt seeding rates as needed, to account for operational differences in agronomic and environmental factors influencing OSR relative to yield (Figure 2).

## Conclusion

Environment and phenotypic characteristics for straw strength and tillering capacity, influence the seeding rate that is optimal for yield in HRSW production. For environments where average yield is ≥3.2 Mg ha^–1^, the OSR is generally higher in comparison to OSR for lower yielding environments (4.5 versus 3.7 million seeds ha^–1^), and when seeding cultivars with high tillering capacity. Adjustments to OSR can also be expected when seeding cultivars with poor straw strength (rating ≥ 5). Breeders and agronomists should utilize this information to focus efforts on characterizing advanced breeding lines and new cultivars for specific genetic and phenotypic traits influencing OSR. Growers can benefit from these findings by adapting seeding rates relative to their average yields; especially when seeding new HRSW cultivars.

## Data Availability Statement

The datasets generated for this study are available on request to the corresponding author.

## Author Contributions

JS drafted the manuscript and was the doctoral student working on the project. GM, JW, and JR edited the draft and provided considerable contributions to the seeding rate dataset.

## Conflict of Interest

GM was employed by the company Bayer CropScience after completion of the 2013–2015 experiments. The remaining authors declare that the research was conducted in the absence of any commercial or financial relationships that could be construed as a potential conflict of interest.
